# Genetics of focal segmental glomerulosclerosis

**DOI:** 10.1007/s00467-007-0445-y

**Published:** 2007-05-01

**Authors:** Robert P. Woroniecki, Jeffrey B. Kopp

**Affiliations:** 1 Albert Einstein College of Medicine, Bronx, NY USA; 2 Kidney Disease Section, National Institute of Diabetes and Digestive and Kidney Disease, National Institutes of Health, Department of Health and Human Services, Bethesda, MD 20892 USA; 3 The Children’s Hospital at Montefiore, 111 East 210th Street, Bronx, NY 10467 USA

**Keywords:** Gene mutations, Filtration barrier, Linkage analysis, Positional cloning

## Abstract

The recent advances in understanding the pathophysiology of focal segmental glomerulosclerosis (FSGS) and molecular function of glomerular filtration barrier come directly from genetic linkage and positional cloning studies. The exact role and function of the newly discovered genes and proteins are being investigated by in vitro and in vivo mechanistic studies. Those genes and proteins interactions seem to change susceptibility to kidney disease progression. Better understanding of their exact role in the development of FSGS may influence future therapies and outcomes in this complex disease.

## Introduction

Focal segmental glomerulosclerosis (FSGS) is not a single disease, as initially thought [1], but the histological expression of a variety of distinct conditions [2]. It is a clinically and genetically heterogeneous entity characterized by a common renal biopsy picture of segmental scarring of the glomerular capillary tuft affecting one or more glomeruli. Immunoglobulin (Ig)M and complement C3 may be present in mesangium, attributed to macromolecular trapping, but other immune deposits are absent. Hyaline deposits may occupy capillary loops, initially in a subendothelial location. In general, FSGS can be divided into three etiologic categories: idiopathic, genetic, and reactive (the latter including postadaptive and medication-associated forms). Although the abnormalities of glomerular filtration barrier have been implicated in the pathophysiology of nephrotic syndrome for the last 30–40 years, it was only during the last decade when genetic linkage studies coupled with positional cloning uncovered new genes and their products that were causally linked to human nephrotic syndrome. Identifying expression abnormalities of those genes contributed to the understanding of the pathophysiology of FSGS in recent years.

Understanding the genetics of FSGS begins with appreciating the molecular composition of glomerular filtration barrier comprised of podocytes, basement membrane (GBM), and fenestrated endothelium. This barrier separates blood from the urinary space and under physiologic conditions selectively permits the ultrafiltration of solutes and preventing the excessive leakage of large molecules, such as albumin and clotting factors, with a molecular weight greater than 40 kDa [3]. The glomerular filtration barrier becomes incompetent and “leaky” in the case of nephrotic syndrome, including FSGS. Under physiologic conditions, podocytes are terminally differentiated epithelial cells with a cell body and several foot processes that are in contact with each other through the interpodocyte connection, called slit diaphragm (SD). All of the genetic defects identified to date affect gene transcription or assembly of critical podocyte functional structures, including SD, actin-based cytoskeleton, and adhesion complexes. These genes include nephrin (*NPHS1*), podocin (*NPHS2*), alpha-actinin-4 (*ACTN-4*), CD2-associated protein (*CD2AP*), Wilm’s tumor gene (*WT1*), and transient receptor potential cation 6 (*TRPC6*) (Table 1). Recently, mutations in phospholipase epsilon C have been identified as a cause of steroid-sensitive nephrotic syndrome [4]. It is still not understood why the FSGS lesion is focal (only some glomeruli are affected) and segmental (only part of the glomerulus has the anatomic lesion).

**Table 1:** Gene mutations that are causally linked to focal segmental glomerulosclerosis (FSGS)

Gene symbol	Gene locus	Protein	Mode of inheritance	Renal manifestations	Extrarenal manifestations
*NPHS2*	1q25.31	Podocin	Autosomal recessive	Minimal change nephropathy, FSGS	None
*ACTN4*	19q13	Alpha-actinin-4	Autosomal dominant	FSGS	None
*TRPC6*	11q21.22	Transient receptor potential cation channel 6	Autosomal dominant	FSGS	None
*PLCE1*	10q23.24	Phospholipase C epsilon	Autosomal recessive	Diffuse mesangial sclerosis and FSGS	None
*WT1*	11p13	Wilm’s tumor suppressor protein	Autosomal dominant, de novo mutation	Diffuse mesangial sclerosis and FSGS	Genitourinary abnormalities
*LMXB1*	9q34.1	Lim homeobox transcription factor 1β	Autosomal dominant	FSGS	Dystrophic nails, absent or malformed patella
*tRNA*^*Leu*^	Mitochondrial genome	NA	Maternal	FSGS, tubulointerstitial nephritis	Muscle and brain disease, lactic acidosis, deafness, diabetes mellitus
*COQ2*	4q21.22	Coenzyme Q2 homolog, prenyltransferase	Autosomal recessive	FSGS	Neurologic and muscle abnormalities
*ITGB4*	17q25.1	Integrin β4	Autosomal recessive	FSGS	Epidermolysis bullosa

## Nephrin (*NPHS1*)

The *NPHS1* gene mutations cause congenital nephrotic syndrome of the Finnish type, an autosomal recessive disorder characterized by massive proteinuria in utero and nephrosis at birth. Although this disease is not pathologically characterized by FSGS, it was the first mutation of structural protein of podocyte causally linked to nephrotic syndrome [5]. Moreover, nephrin deficiency was detected in other forms of nephrotic syndrome, and an overlap in the genes encoding nephrin/podocin (*NPHS1/NPHS2*) mutation spectrum (a triallelic hit) has recently been documented in patients with congenital FSGS [6]. *NPHS1* product nephrin has 1,241 amino acid residues and belongs to the immunoglobulin (Ig) family of cell adhesion molecules. It contains a transmembrane domain, eight Ig-like repeats, and one fibronectin III-like module. Nephrin was localized by immunogold labeling and electron tomography at the slit between podocyte foot processes in a network of 35-nm long, globular cross-strands lining lateral, elongated pores in a zipper-like model [7]. Those pores are the same as or smaller than albumin molecules. Patients with *NPHS1* mutations and nephrin-knockout mice had only narrow filtration slits that lacked the slit diaphragm network and the 35-nm-long strands; these findings suggest that nephrin molecules are directly involved in constituting the macromolecule-retaining slit diaphragm and its pores [8]. Functionally, nephrin controls the podocyte cytoskeleton in vivo by interacting with other proteins, such as Src homology-2 (SH2)/SH3 domain-containing Nck adaptor proteins [7]. Originally described mutation in Finnish patients was a two-nucleotide deletion in exon 2 [5], but other mutations have been reported since. Those include deletions, insertions, nonsense, missense, splicing mutations, and common polymorphisms [9–11].

## Podocin (*NPHS2*)

Positional cloning identified *NPHS2*, encoding podocin, as a causative gene in autosomal recessive steroid-resistant nephrotic syndrome, including FSGS [12]. *NPHS2* encodes a putative 383 amino acid protein of approximately 42 kDa. It belongs to the stomatin family proteins (band-7 proteins). *NPHS2* expression is restricted to the podocytes, as shown by in situ hybridization studies, and the protein is an integral plasma membrane protein associated with specialized lipid raft microdomains [12]. Podocin is required for the recruitment of nephrin into lipid rafts; it interacts with nephrin and CD2AP [13] and facilitates nephrin signaling [14]. In podocin-null mice, nephrin expression is increased, whereas ZO1 (tight junction protein) and CD2AP expression is decreased. The mice die a few days after birth from renal failure caused by diffuse mesangial sclerosis, and they develop proteinuria during the antenatal period [15].

At present, at least 26 different *NPHS2* mutations associated with FSGS have been described, with a mutation defined as a polymorphism that is present in homozygosity in at least one patient with FSGS [12, 16–20]. Many patients are compound heterozygotes with two distinct mutations. The age of onset is generally in infancy or childhood, but a few cases of adult onset kidney disease have been described, with the oldest patient presenting at age 36. In populations of European ancestry, *NPHS2* mutations are present in 26% of families with familial FSGS and 12–19% of sporadic pediatric FSGS [16, 18]. On the other hand, *NPHS2* mutations associated with pediatric FSGS appear to be uncommon in children of Asian ancestry [21]; little is known about children of African ancestry. FSGS associated with *NPHS2* mutations is uniformly steroid resistant and generally shows poor response to cyclosporine as well [18]. Although early reports suggested otherwise, the consensus is now that patients with *NPHS2* mutations are at reduced risk for recurrent FSGS following renal transplant [18, 22]. The most common polymorphism is *R229Q*, with a heterozygote frequency in the general population ranging from 0.03 to 0.13 [23]. *R229Q* heterozygosity has been associated with microalbuminuria in a Brazilian population [24] and a modest increased risk for FSGS in individuals of European ancestry, but not individuals of African ancestry [23].

## CD2-associated protein (*CD2AP*)

CD2-associated protein was discovered as a binding ligand to the adhesion molecule CD2 during cell–cell interaction of lymphocyte T with antigen presenting cell. This interaction initiates the process of protein segregation, CD2 clustering, and cytoskeletal polarization. The *CD2AP* gene encodes a protein of 639 amino acids with a deduced molecular mass of approximately 70 kDa, and messenger ribonucleic acid (mRNA) is ubiquitously expressed in human tissues. *Cd2ap* knock-out mice manifest compromised immune function and they developed proteinuria by 2 weeks of age and died from renal failure at 6–7 weeks of age [25]. In the kidney, the lesion resembles FSGS, with mesangial cell hyperplasia and extracellular matrix deposition. Mice with *Cd2ap* haploinsufficiency (heterozygous deletion) develop glomerular changes at 9 months of age. They have increased susceptibility to glomerular injury by immune complexes, with likely impairment of the intracellular degradation pathways [26]. CD2AP is the human ortholog of mouse Cd2ap, with 86% identity at the amino acid level. CD2AP (originally named CMS) was identified in the yeast two-hybrid system as interacting partner of p130 (Cas), a cell cycle regulatory protein [27]. CD2AP is a multifunctional adapter molecule, which is localized to the cytoplasm, membrane ruffles, and leading edges of cells. It functions as scaffolding protein involved in the dynamic regulation of the actin cytoskeleton [27]. Two African Americans with primary idiopathic FSGS were found to have a mutation of *CD2AP* splice acceptor of exon 7 on one allele. This mutation was not seen in control subjects. Although these data are suggestive, family studies showing a consistent relationship between mutation and renal phenotype will be required to establish a pathogenic role. Data supporting a role for CD2AP haploinsufficiency comes from bigenic mouse models of FSGS involving pairwise interaction of CD2AP, Fyn, and synaptopodin [28].

## Alpha-actinin-4 (*ACTN4*)

*ACTN4* mutations have been found to be the cause of autosomal dominant FSGS in five families [29, 30]. These patients tend to present in the teenage years or later and progress to end-stage renal disease (ESRD) slowly. The penetrance is variable, and some patients with mutations have a very mild phenotype. Alpha-actinin-4 is an actin-bundling protein associated with cytoskeleton, cell motility, and cancer invasion [31]. It is highly expressed in podocytes, and interacts with synaptopodin and with the tight junction protein membrane-associated guanylate kinase (MAGI)-1 in rat kidney epithelial cells [32]. Mutant alpha-actinin-4 binds actin more tightly that does wild-type alpha-actinin-4, indicating a gain of function mutation. *Actn4*-null mice develop proteinuria, progressive glomerular disease, and die by several months of age. Histological abnormalities are limited to the kidneys and include diffuse podocyte foot process effacement and globally disrupted morphology [33]. Cultured podocytes from these mice adhere poorly to basement membrane components, suggesting that podocyte loss may contribute to podocytopenia and FSGS [34].

## Transient receptor potential cation channel (*TRPC6*)

TRPC6 belongs to a protein family whose members function as ion channels, mediating capacitative calcium entry into the cell. TRPC6 is a nonselective calcium channel that is activated by diacylglycerol in a membrane-delimited fashion, independently of protein kinase C [35]. TRPC6 is responsible for calcium entry during cell proliferation and is expressed primarily in the placenta, lung, spleen, ovary, and small intestine. In the kidney, TRPC6 is expressed in tubules and glomeruli, including podoctyes and glomerular endothelial cells.

*TRPC6* mutations have been identified in six families of European and African geographic ancestry with autosomal dominant FSGS [36, 37]. Each family carried a distinct missense mutation. Age at renal disease presentation ranged from 17 to 52 years, and the duration from onset to ESRD averaged approximately 10 years. TRPC6 interacts with nephrin and podocin, thus localizing the protein to the slit diaphragm complex. Two mutant proteins are associated with increased calcium amplitudes, indicating an activating mutation. The discovery that abnormal TRPC6 disturbs podocyte function suggests that calcium signaling may play a critical role in facilitating podocyte regulation of intracellular function, including control of cytoskeletal and foot process architecture.

## Phospholipase C epsilon 1 (*PLCE1*)

Recently, seven families with infantile or early onset proteinuric renal disease were found to have mutations in *PLCE1*, which encodes an isoform of phospholipase C, an enzyme that participates in intracellular signaling [4]. In affected individuals, proteinuria was detected between age 2 months and 9 years; renal histology included diffuse mesangial sclerosis and FSGS. Response to treatment included steroid resistance and complete remission to either steroids or cyclosporine. This is the first time a genetic podoctye disease has been associated with steroid-sensitive nephrotic syndrome [4].

## Syndromic FSGS

The above genes have mutations associated with FSGS in the absence of extrarenal manifestations. To date, at least five genes have been associated with syndromes of which FSGS is often a part.

### *WT1*

The product of the Wilm’s tumor gene *(WT1)* is required for normal development of the genitourinary system and mesothelial tissues and is overexpressed in leukemia and various types of solid tumors. *WT1* mutations have been identified in patients with Wilm’s tumor, WAGR syndrome (Wilm’s tumor, aniridia, genitourinary abnormalities, and retardation-hypospadias and bilateral cryptorchidism may be seen), Denys-Drash syndrome (urogenital abnormalities, renal failure, pseudohermaphroditism, and Wilm’s tumor), Frasier syndrome (male pseudohermaphroditism and progressive glomerulopathy), and isolated diffuse mesangial sclerosis. *WT1* has four major RNA splice variants, and the interactions between the four polypeptide products may play a role in the control of cellular proliferation and differentiation. WT1 acts as either a transcriptional activator or repressor, depending on chromosomal and cellular context. WT1 is expressed predominantly in the kidney and certain hematopoietic cells [38].

The Wilm’s tumor upstream neighbor (*WIT1*) is located upstream of *WT1*, localized to chromosome 11p13. *WIT1* is transcribed in the opposite direction of *WT1*, and some *WIT1* splice variants include antisense portions of WT1. *WIT1* transcripts may play a role in transcriptional regulation of *WT1* [39]. WIT1 protein contains 92 amino acids and is expressed in fetal kidney and spleen [40]. Single nucleotide polymorphisms (SNPs) in both *WT1* and *WIT1* and their common promoter (rs6508, rs2301254, and rs1799937) were significantly associated with HIV-seronegative FSGS, suggesting that variants in these genes may mediate pathogenesis by altering WT1 function [41].

### *LMX1B*

The nail–patella syndrome includes various combinations of dystrophic nails, absent or malformed patellas, elbow contractures, glaucoma, and FSGS [42]. The gene responsible, *LMX1B*, is a transcription factor required for expression of *CD2AP* and *NPHS2*, among other genes [43].

### *tRNA*^Leu^

Mutations in the mitochondrial gene encoding tRNA^Leu^ are associated with the MELAS syndrome (*m*yopathy, *e*ncephalopathy, *l*actic acidosis, and *s*troke-like episodes), typically presenting in infancy or early childhood. There is a maternal inheritance pattern, and the most common mutation is A324G [44, 45]. Patients with this mutation may also present later in childhood or in adulthood with FSGS associated with deafness or diabetes mellitus, or occasionally with sporadic FSGS.

### *COQ2*

Another mitochondriopathy associated with FSGS has been described recently. A mutation in *COQ2*, encoding an enzyme required for synthesis of coenzyme Q_10_ (ubiquinone), which is present in all membranes. A *COQ2* mutation was found in an infant who presented at 12 months of age with psychomotor delay, optic atrophy, and FSGS [46].

### *ITGB4*

An infant with epidermolysis bullosa, pyloric atresia, and FSGS was found to have a homozygous missense mutation (R1281W) in β4 integrin. The α6β4 integrin binds laminin-5 (a trimer composed of the laminin α5, β3, and γ3 chains). Although the major podocyte integrin is α3β1, the authors showed that podocytes do express low levels of α6β4. Whereas other families with α6β4 integrin mutations and epidermolysis bullosa have been reported, to date, only the one case of FSGS has been reported [47].

## Summary

The recent advances in understanding the pathophysiology of FSGS and molecular function of glomerular filtration barrier come in large part from genetic linkage and positional cloning studies. Because a substantial percentage (up to 30%) of sporadic, steroid-resistant nephrotic syndrome patients carry the specific genetic mutation causally linked to FSGS, it may be advisable that all patients who have failed steroids should be offered genetic screening for mutations at the time of or prior to starting more aggressive treatments, as those patients have been shown to unfavorably respond to immunosuppressant therapy [18]. Genetic testing could be performed in several research laboratories and now is also being offered by a commercial laboratory. Genetic testing for familial forms of FSGS might also be clinically informative in situations where living-related donor transplant is considered.

The exact role and function of the newly discovered genes and proteins are being investigated by in vitro and in vivo mechanistic studies. Those genes and proteins interactions seem to change susceptibility to kidney disease progression. Better understanding of their exact role in the development of FSGS may influence future therapies and outcomes in this complex disease.

### Questions

(Answers appear following the reference list)

1. Familial form of FSGS is most commonly associated with mutation of:
  - *NPHS1* (gene encoding nephrin)
  - *WT1* (gene encoding Wilm’s tumor protein)
  - *CD2AP* (gene encoding CD2-associated protein)
  - *NPHS2* (gene encoding podocin)
2. Mutation of which gene is likely associated with steroid-sensitive nephrotic syndrome:
  - *NPHS1*
  - *NPHS2*
  - *TRPC6*
  - None of the above
3. Although established as a cause and model of FSGS in animals, mutation of which gene is only rarely found in humans:
  - *NPHS1*
  - *NPHS2*
  - *CD2AP*
  - *TRPC6*
4. Proteins that interact with each other in the vicinity of the slit diaphragm are:
  - Nephrin, WT1, TRPC6
  - Nephrin, Alpha-actinin-4, Podocin
  - Podocin, CD2AP, Nephrin
  - Nephrin, CD2AP, Alpha-actinin-4
5. FSGS that is associated with hypospadias and bilateral cryptorchidism may result from the mutation of:
  - *NPHS2*
  - *WT1*
  - *CD2AP*
  - *TRPC6*

### Answers

1. D
2. D
3. C
4. C
5. B
